# Correction: Light-induced assembly and disassembly of polymers with Pd_*n*_L_2*n*_-type network junctions

**DOI:** 10.1039/d1sc90054d

**Published:** 2021-03-18

**Authors:** Ru-Jin Li, Cristian Pezzato, Cesare Berton, Kay Severin

**Affiliations:** Institut des Sciences et Ingénierie Chimiques, École Polytechnique Fédérale de Lausanne (EPFL) 1015 Lausanne Switzerland kay.severin@epfl.ch

## Abstract

Correction for ‘Light-induced assembly and disassembly of polymers with Pd_*n*_L_2*n*_-type network junctions’ by Ru-Jin Li *et al.*, *Chem. Sci.*, 2021, DOI: 10.1039/d1sc00127b.

The authors regret that the structure of the cyclised photoacid in Scheme 1 and in the Graphical Abstract contained a mistake where a N^+^=C bond was shown instead of a N–C bond. The correct version of Scheme 1 is shown below and the Graphical Abstract has also been corrected.

**Scheme 1:**
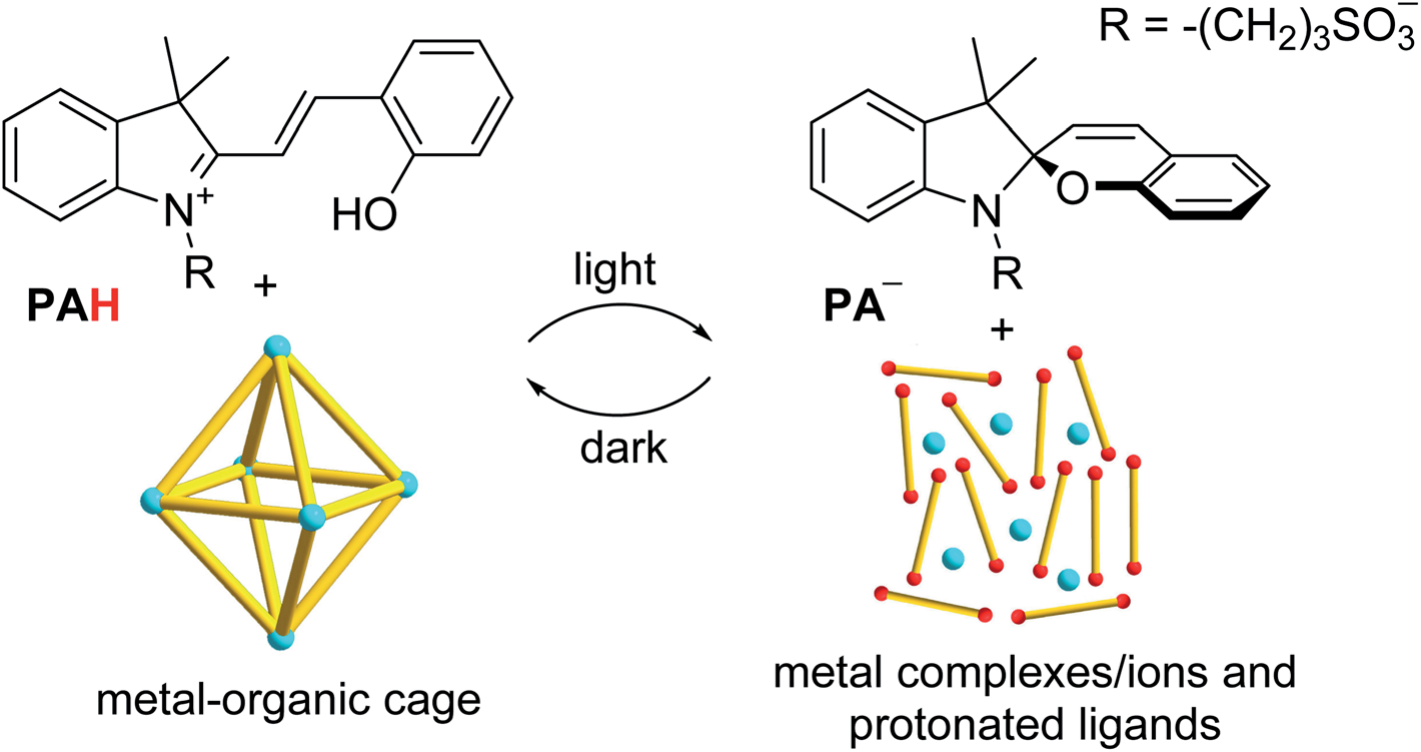
The photoacid **PAH** allows controlling the assembly of metal–organic cages.

The authors also regret that the direction of the reaction arrows between the open and closed form of the photoacid in Fig. S32 of the ESI was previously incorrect, and unrelated ref. S12–S14 about crystallographic work were listed. Fig. S32 has now been corrected in the ESI, and the previous ref. S12–S14 have been removed.

The Royal Society of Chemistry apologises for these errors and any consequent inconvenience to authors and readers.

